# Preparation and Characterization of Epoxy Resin Filled with Ti_3_C_2_T_x_ MXene Nanosheets with Excellent Electric Conductivity

**DOI:** 10.3390/nano10010162

**Published:** 2020-01-17

**Authors:** Ailing Feng, Tianqi Hou, Zirui Jia, Yi Zhang, Fan Zhang, Guanglei Wu

**Affiliations:** 1 Institute of Physics & Optoelectronics Technology, Baoji University of Arts and Sciences, Baoji 721016, China; ailing@mail.xjtu.edu.cn; 2 Institute of Materials for Energy and Environment, State Key Laboratory of Bio-Fibers and Eco-Textiles, College of Materials Science and Engineering, Qingdao University, Qingdao 266071, China; qdhoutq@163.com (T.H.); zhangy@njtech.edu.cn (Y.Z.); 3 School of Materials Sciences and Engineering, East China Jiaotong University, Nanchang 330013, China; 4 Key Laboratory of Engineering Dielectrics and Its Application, Ministry of Education, Harbin University of Science and Technology, Harbin 150080, China

**Keywords:** MXene, epoxy resin, electrical conductivity, mechanical properties

## Abstract

MXene represents new kinds of two-dimensional material transition metal carbides and/or carbonitrides, which have attracted much attention in various applications including electrochemical storage devices, catalysts, and polymer composite. Here, we report a facile method to synthesize Ti_3_C_2_T_x_ MXene nanosheets and prepare a novel electrically conductive adhesive based on epoxy resin filled with Ti_3_C_2_T_x_ MXene nanosheets by solution blending. The structure, morphology, and performance of Ti_3_C_2_Tx MXene nanosheets and epoxy/Ti_3_C_2_T_x_ MXene nanosheets composite were investigated. The results show that Ti_3_C_2_T_x_ MXene possesses nanosheet structure. Ti_3_C_2_T_x_ MXene nanosheets were homogeneously dispersed in epoxy resin. Electrical conductivity and mechanical properties measurements reveal that the epoxy/Ti_3_C_2_T_x_ MXene nanosheet composite exhibited both good electrical conductivity (4.52 × 10^−4^ S/m) and favorable mechanical properties (tensile strength of 66.2 MPa and impact strength of 24.2 kJ/m^2^) when the content of Ti_3_C_2_T_x_ MXene nanosheets is 1.2 wt %. Thus, Ti_3_C_2_T_x_ MXene is a promising filler for electrically conductive adhesive with high electric conductivity and high mechanical performance.

## 1. Introduction

With the progression of science and technology, electronic units are developing towards the direction of miniaturization, portability, and high integration [1,2,3,4,5]. Therefore, the electronic packaging industry demands interconnect bonding technology, which could offer excellent electrical conductivity, good flexibility, and satisfied mechanical strength [6,7,8,9]. Besides, it should be cost effective and environmentally benign. In this regard, electrical conductive adhesives (ECAs) are regarded as one of the most feasible alternative interconnection materials for future applications due to low processing temperature, fine pitch interconnect, and environmental friendliness [10,11,12,13].

ECAs consist of polymer (such as, acrylate resin, silicone, or epoxy resin) and electrical conductive fillers (such as, Ag, Au, Ni, or carbon materials). The polymer resin provides interconnect bonding properties while conductive fillers conduct electricity in ECAs. This polymer is widely used because of its low density, high specific strength, dimensional stability, and chemical resistance [14,15,16,17,18]. Many researches have studied epoxy-based ECAs [19]. The formation of effective conductive paths in the ECAs is the key to obtain high electrical conductivity. Several conductive fillers have been studied in ECAs; these include cobalt nanoparticles [20], zinc complexes [21,22], cadmium [23], metal oxide [24], and so on. However, there are some issues such as aggregation and poor interconnect between conductive fillers with polymer resin, which need to be solved.

MXenes is a new kind of transition metal carbide/nitride two-dimensional material with graphene-like structure. Their chemical formulas are M_n+1_X_n_, where M is an early transition metal, X is C and/or N, and n represents the number of X (1, 2, or 3). MXenes are promising conductive fillers due to intrinsic high conductivity and unique structure [25,26,27]. Carbon materials (graphene or carbon nanotube) have to be oxidized to introduce oxygen-containing functional groups, which prevent carbon materials aggregating in the polymer resin and assist binding between graphene/carbon nanotube and EP resin. However, the appearance of some functional groups decreases the electronic conductivity. MXene does not require any pretreatment to composite with other materials, as the surface atomic of MXenes is Ti, which can easily bind the O atomic of EP resin. Therefore, MXenes could well be dispersed in the polymer resin and provide high electric conductivity.

Herein, we report on the structure and performance of EP/MXene ECAs. Ti_3_AlC_2_ was used as the MAX precursor to prepare Ti_3_C_2_T_x_ MXene nanosheets. The crystal structure and morphology of Ti_3_C_2_T_x_ MXene nanosheets were characterized by scanning electron microscope (SEM), X-ray diffraction (XRD), and transmission electron microscopy (TEM). Different amounts of Ti_3_C_2_T_x_ MXene nanosheets were introduced into ECAs to study the effect of Ti_3_C_2_T_x_ MXene nanosheets on the electrical properties and mechanical performance of the EP/Ti_3_C_2_T_x_ MXene nanosheet ECAs. The results show that the electrical property and mechanical performance of the EP/Ti_3_C_2_T_x_ MXene nanosheets ECAs could be improved compared to pure EP resin. Our work may shed lights on the development of ECAs with high electric conductivity and high mechanical performance in the electronic industry.

## 2. Experimental

### 2.1. Material

Epoxy resin and hardener tetrahyelrophthalic anhydride were purchased from Sigma-Aldrich. The titanium carbide (TiC) powders were obtained from Alfa Aesar. Commercially available Ti_2_AlC was obtained from Kanthal Co., Ltd., Stockholm, Sweden; 36% hydrochloric acid (HCl) and lithium fluoride (LiF, 98%) were bought from Sinopharm Chemical Reagent Co., Ltd., Shanghai, China.

### 2.2. Preparation of EP/Ti_3_C_2_T_x_ MXene Nanosheets ECAs

The Ti_3_AlC_2_ was prepared by mixing Ti_2_AlC and TiC powders in a 1:1 M ratio, followed by ball-milling at 300 rpm for 18 h. After being milled, the mixture was heated to 1350 °C at 5 °C/min in an Al_2_O_3_ tube furnace under Ar atmosphere. Then, the mixture was reacted at 1350 °C for 2.0 h before cooling again. The resulting product is Ti_3_AlC_2_.

First, 0.66 g LiF was added in 6 M HCI solution; 1.0 g Ti_3_AlC_2_ powders were immersed in 50 mL LiF/HCl solution. The mixture was stirred for 48 h at 40 °C. The resulting suspension was washed using distilled water five times and centrifuged to separate the powder from the supernatant. Then, the obtained powder was sonicated for 2.0 h and rinsed three times with deionized water. Ti_3_C_2_T_x_ MXene nanosheets were collected and dried in vacuum at 80 °C for 24 h.

**Figure 1:**
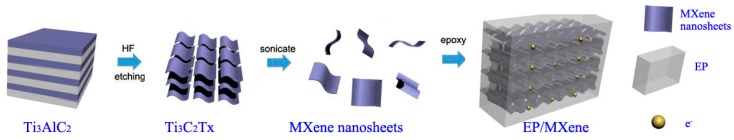
The schematic illustration for the synthetic procedure of the EP/Ti_3_C_2_T_x_ MXene nanosheets composites.

The resin mixture was uniformly mixed at the epoxy resin and curing agent (methyl tetrahyelrophthalic anhydride) mass ratio equal to 100:32. Then, different amounts of Ti_3_C_2_T_x_ MXene nanosheets added into resin mixture. To achieve uniform dispersion of Ti_3_C_2_T_x_ MXene nanosheets in the epoxy resin, the mixture was mixed with a Flacktek Speed mixer at a speed of 2000 rpm for 0.5 h. The curing process took place at 100 °C for 2 h, 120 °C for 2 h and 160 °C for 4 h. After cooling to room temperature, the resulting EP/Ti_3_C_2_T_x_ MXene nanosheets composites were obtained. The schematic illustration of preparation of EP/Ti_3_C_2_T_x_ MXene nanosheets composites is illustrated in Figure 1.

### 2.3. Characterization and Measurement

The surface morphology of Ti_3_AlC_2_ and Ti_3_C_2_T_x_ MXene nanosheets and the fracture surfaces of pure EP, EP/Ti_3_C_2_T_x_ MXene nanosheets composites were characterized by SEM (FEI, Quanta 250, Washington, WA, USA). The element contents in Ti_3_C_2_T_x_ MXene nanosheets were characterized by energy-dispersive X-ray (EDX, FEI, Quanta 250). The phase structures of Ti_3_AlC_2_ and Ti_3_C_2_T_x_ MXene were characterized by X-ray powder diffraction (XRD, Rint-2000, Rigaku, Berlin, Germany) using Cu Kα radiation (λ = 1.5418 Å) operated at a voltage of 30 kV and a current of 10 mA. Fourier transform infrared spectrum (FTIR, IRpresitge-21 model, Tokyo, Japan) was used to record the Fourier transform infrared spectra of Ti_3_AlC_2_ and Ti_3_C_2_T_x_ MXene. The internal morphology of Ti_3_C_2_T_x_ MXene nanosheets were studied using transmission electron microscope (TEM, JEOL, JEM-2100 HT, Tokyo, Japan). There are four methods (Volt-Ampere, double bridge, direct current four probe, and digital multimeter) to measure the conductivity of composites. We used digital multimeter (830/F-33 model, Beijing Jiaxin Instrument, Ltd., Beijing, China) to test the resistance (*R*) of EP/Ti_3_C_2_T_x_ MXene nanosheets composites. Composite circles with a diameter of 20 mm and a thickness of 2 mm were prepared. Then we calculated the resistivity (*ρ*) of composite based on the diameter and thickness of samples. Electrical conductivity (*K*) is the reciprocal of resistivity (*ρ*) as follows: *K* = 0.0637/*R* (S/cm). The mechanical properties of EP/Ti_3_C_2_T_x_ MXene nanosheets composites were tested using tensile testing machine (UTM-2460 model, Hebei JInjian Co., Ltd., Tangshan, China).

## 3. Results and Discussion

### 3.1. The Structure and Morphology of Ti_3_AlC_2_ and Ti_3_C_2_T_x_ MXene

**Figure 2:**
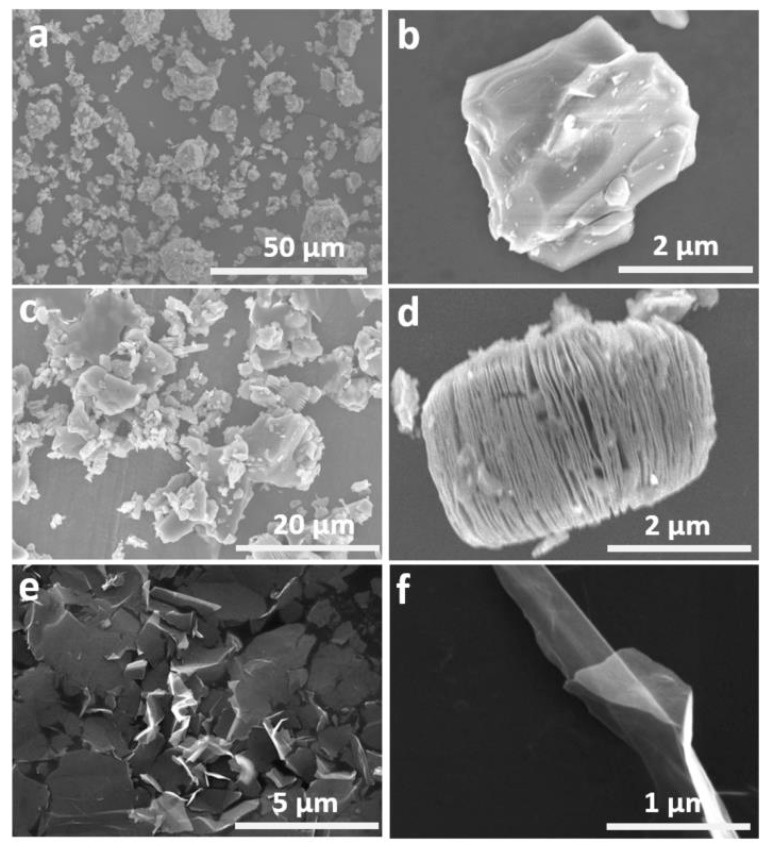
SEM images of Ti_3_AlC_2_ MAX (**a**) at a lower magnification, (**b**) at a higher magnification; SEM images of accordion-like multi-layered Ti_3_C_2_T_x_ (**c**) at a lower magnification, (**d**) at a higher magnification); SEM images of Ti_3_C_2_T_x_ nanosheets (**e**) at a lower magnification, and (**f**) at a higher magnification).

The SEM images of Ti_3_AlC_2_, Ti_3_C_2_T_x_, and Ti_3_C_2_T_x_ nanosheets are shown in Figure 2. SEM images (Figure 2a,b) show that Ti_3_AlC_2_ display a solid bulk morphology with uneven sizes. The smaller Ti_3_AlC_2_ MAX is about 2 μm while the larger one is about 20 μm. After HF etching, Ti_3_C_2_T_x_ show a loosely packed structure consist of multilayered Ti_3_C_2_T_x_ nanosheets, which had typical diameters of 2 μm (Figure 2c,d). The interlayer space of multilayered Ti_3_C_2_T_x_ is ~200 nm. This morphology is because the LiF/HF treatment removes Al layers from Ti_3_AlC_2_ MAX. By sonication, the loosely packed Ti_3_C_2_T_x_ MXene is converted to nanosheets (Figure 2e,f). The Ti_3_C_2_T_x_ MXene nanosheets have smooth surface, which show a structure similar to that of graphene. The thin sheet is of ~10 nm thick, which corresponds to roughly 5 Ti_3_C_2_T_x_ layers.

**Figure 3:**
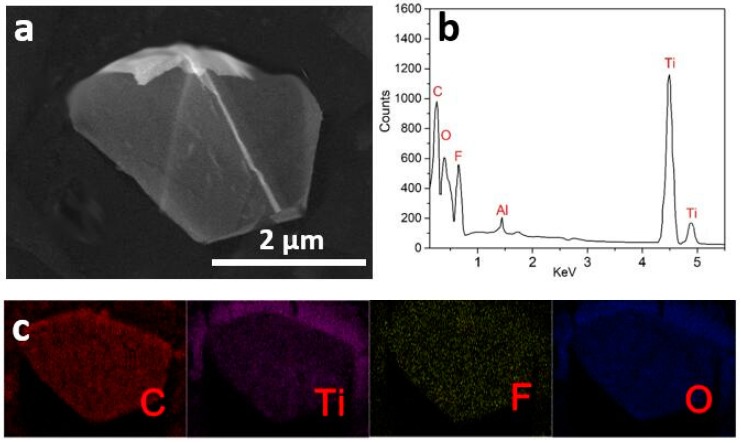
(**a**) SEM image of Ti_3_C_2_T_x_ nanosheets, (**b**) energy-dispersive X-ray spectroscopy (EDX) spectrum of Ti_3_C_2_T_x_ nanosheets, and (**c**) element mappings of Ti_3_C_2_T_x_ nanosheets.

To study the elements of Ti_3_C_2_T_x_ nanosheets, energy dispersive X-ray (EDX) analysis were carried out. Figure 3 reveal EDX spectrum and SEM-EDX element mappings of Ti_3_C_2_T_x_ MXene nanosheets. As shown in Figure 3a, Ti_3_C_2_T_x_ MXene thin nanosheets have diameters of 3 μm. In the EDX spectrum (Figure 3b), four strong peaks are belonged to C, Ti, O, and F, whereas a weak peak assigns to Al. As shown in Figure 3c, the uniform C, Ti, O, and F elemental distributions are observed. The composition of Ti_3_C_2_T_x_ nanosheets, as calculated from EDS analysis, is shown in Table 1. The results showed that the Ti_3_C_2_T_x_ MXene contains 11.0 wt % C, 69.0 wt % Ti, 11.1 wt % O, 7.8 wt % F and 1.1 wt % Al. The F and O elements are attributed to presence of –F, –OH, and –COOH.

**Figure 4:**
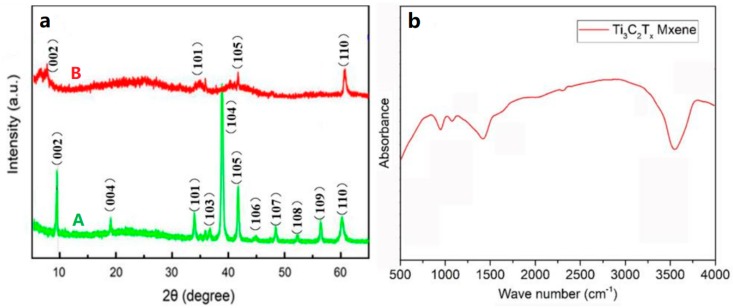
(**a**) XRD patterns of Ti_3_AlC_2_ MAX (A) and Ti_3_C_2_T_x_ nanosheets (B). (**b**) FTIR spectra of Ti_3_C_2_T_x_ nanosheets.

The structural properties of Ti_3_AlC_2_ and Ti_3_C_2_T_x_ MXene were tested using XRD and Fourier transform infrared spectroscopy (FTIR), as shown in Figure 4. The diffraction pattern (Figure 4aA) has eleven broad peaks at 9.5°, 19.0°, 34.0°, 36.8°, 38.8°, 41.9°, 45.1°, 48.5°, 52.7°, 56.6°, and 60.2°, corresponding to (002), (004), (101), (103), (104), (105), (106), (107), (108), (109), and (110) of Ti_3_AlC_2_ MAX (JCPDS No. 52-0875) [28,29], respectively. It can be seen in the XRD patterns that the crystallinity and structural order of Ti_3_AlC_2_ decrease after HF treatment. As shown in Figure 4aB, no characteristic diffraction peaks of at 2θ = 36.9°, 38.9°, 42.0° belonged to Ti_3_AlC_2_ are observed (Figure 4aA) because of the removal of Al layers from Ti_3_AlC_2_ MAX. Moreover, the characteristic (002) peak at 2θ = 9.5° shifted to a lower value (2θ = 8.3°) and became broadened, displaying an increase in c-spacing when the Al atoms are removed away and replaced by –F and –OH. The FTIR spectra of Ti_3_AlC_2_ MAX and Ti_3_C_2_T_x_ MXene nanosheets are shown in Figure 4b. There is no obvious peak in Ti_3_AlC_2_ MAX curve, which means there is no organic functional groups on Ti_3_AlC_2_ MAX. From Figure 4b, the obvious band at 3490 cm^−1^ is indicative of the presence of obvious strongly hydrogen-bonded –OH on the surface of Ti_3_C_2_T_x_ MXene nanosheets. A weak peak at 1216 cm^−1^ can be observed. This weak peak is a characteristic of covalent C–F bond stretching vibrations [30]. FTIR result confirmed that Al atoms are replaced by –F, –OH, and –COOH functional groups, which is consistent with the XRD result.

**Figure 5:**
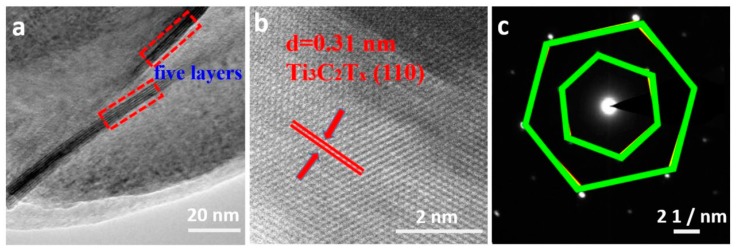
(**a**) TEM image of Ti_3_C_2_T_x_ nanosheets, (**b**) HRTEM image of Ti_3_C_2_T_x_ nanosheets, and (**c**) selected-area electron diffraction (SAED) of Ti_3_C_2_T_x_ nanosheets.

Figure 5 show TEM image and the corresponding selected-area electron diffraction (SAED) of Ti_3_C_2_T_x_ MXene nanosheets. Figure 5a indicates a typical low-magnification TEM image of the synthesized Ti_3_C_2_T_x_ MXene, in which thin nanosheet morphology formed, and the thin nanosheet has five layers, which is consistent with the SEM result. HRTEM was used to characterize the Ti_3_C_2_T_x_ MXene nanosheets (Figure 5b). The Ti_3_C_2_T_x_ MXene sample shows the characteristic spacings of 0.31 nm for the (110) lattice planes of Ti_3_C_2_T_x_ [31]. The SAED pattern of Ti_3_C_2_T_x_ MXene nanosheets showed a hexagonal arrangement at the atomic scale, revealing the typical hexagonal crystalline structure of Ti_3_C_2_T_x_ phase.

### 3.2. The Structure of EP/Ti_3_C_2_T_x_ MXene Nanosheets ECAs

**Figure 6:**
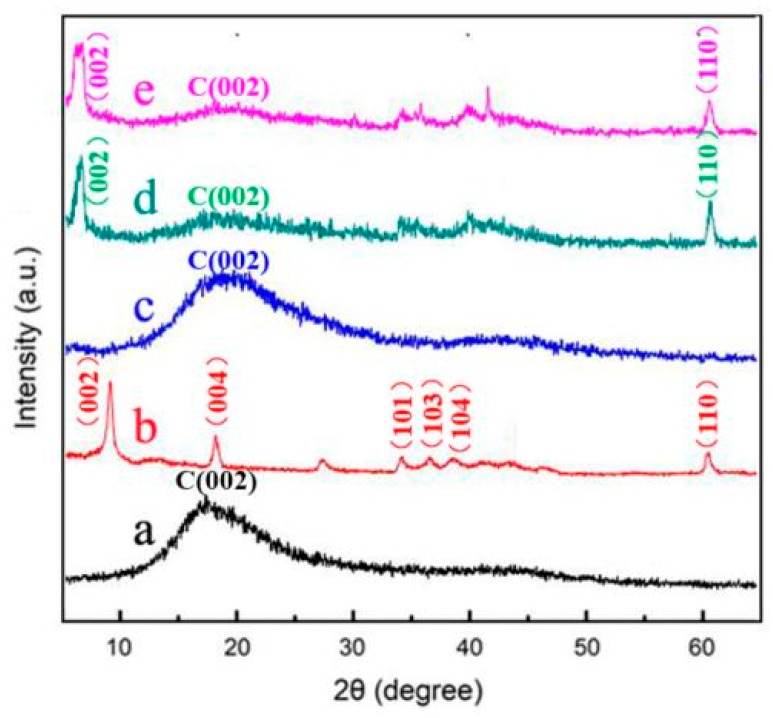
XRD patterns of epoxy (**a**), Ti_3_C_2_T_x_ MXene nanosheets (**b**), EP/Ti_3_C_2_T_x_ MXene nanosheets composite containing 0.4 wt % Ti_3_C_2_T_x_ (**c**), EP/Ti_3_C_2_T_x_ MXene nanosheets composite containing 1.0 wt % Ti_3_C_2_T_x_ (**d**), and EP/Ti_3_C_2_T_x_ MXene nanosheets composite containing 1.6 wt % Ti_3_C_2_T_x_ (**e**).

Figure 6 shows XRD patterns of the epoxy (a), Ti_3_C_2_T_x_ MXene nanosheets (b), EP/Ti_3_C_2_T_x_ MXene nanosheets composite containing 0.4 wt % Ti_3_C_2_T_x_ (c), EP/Ti_3_C_2_T_x_ MXene nanosheets composite containing 1.0 wt % Ti_3_C_2_T_x_ (d), and EP/Ti_3_C_2_T_x_ MXene nanosheets composite containing 1.6 wt % Ti_3_C_2_T_x_ (e). The epoxy has a broad peak at 2θ = 16.8°, whereas Ti_3_C_2_T_x_ MXene nanosheets have peaks at 2θ = 8.8°, 60.2° belonged to (002) and (110) planes. For EP/Ti_3_C_2_T_x_ MXene nanosheets composite, the characteristic (002) peak of Ti_3_C_2_T_x_ MXene nanosheets shifted to a lower value. The EP composite containing 0.4 wt % filler did not give any discernible (002) or (110) peaks (Figure 6c), most likely because the Ti_3_C_2_T_x_ loading was not sufficient to yield any signal. At a higher loading fraction of 1.0 wt %, the composite had the (110) peak and also a similar (002) peak shift. The EP composite containing 1.6 wt % still had the (110) peak which become broad. Explanation for this might be that EP polymer molecular chains are incorporated in the Ti_3_C_2_T_x_ MXene layers which make the d-spacing increase.

### 3.3. Electrical Conductivity and Mechanical Performance

**Figure 7:**
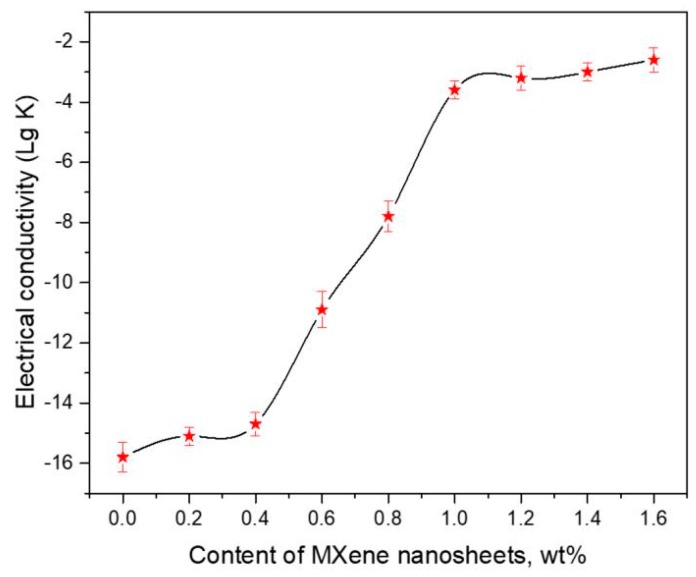
The electrical conductivity (K, S/m) of the EP/Ti_3_C_2_T_x_ MXene nanosheets composite under various mass fractions of Ti_3_C_2_T_x_ MXene nanosheets.

The electrical conductivity (K) of the EP/Ti_3_C_2_T_x_ MXene nanosheets composite under various mass fractions of Ti_3_C_2_T_x_ MXene nanosheets is illustrated in Figure 7. From Figure 7 we can observe that the electrical conductivity of the EP/Ti_3_C_2_T_x_ MXene nanosheets composite increases gradually as the increasing of Ti_3_C_2_T_x_ MXene nanosheets filler content. When the content of Ti_3_C_2_T_x_ MXene nanosheets is lower than 0.4 wt %, the electrical conductivity slowly increases with increasing content of Ti_3_C_2_T_x_ MXene nanosheets. When the content of Ti_3_C_2_T_x_ MXene nanosheets increases to 0.6 wt %, the electrical conductivity of the EP/Ti_3_C_2_T_x_ MXene nanosheets composite increases significantly. The critical content of filler is regarded as the percolation threshold. Thus the percolation threshold of the EP/Ti_3_C_2_T_x_ MXene nanosheets composite is ~0.85 wt %. The electrical conductivity of the EP/Ti_3_C_2_T_x_ MXene nanosheets composite in percolation threshold is 1.81 × 10^−6^ S/m, that is, ten orders of magnitude higher than that of the pure epoxy resin (about 1.03 × 10^−16^ S/m). After that, the electrical conductivity of the EP/Ti_3_C_2_T_x_ MXene nanosheets composites also increases as the content of Ti_3_C_2_T_x_ MXene nanosheets increases. However, its electrical conductivity increases slowly.

The percolation theory was applied for the calculation of the percolation threshold of composites. The dependence of the electrical conductivity of EP/Ti_3_C_2_T_x_ MXene nanosheets composite on Ti_3_C_2_T_x_ MXene content can be described in *σ_m_* = *σ_n_*(*P-Pc*)*^β^*, where *σ_n_* is the conductivity of Ti_3_C_2_T_x_ MXene nanosheets, *σ_m_* is the electrical conductivity of EP/Ti_3_C_2_T_x_ MXene nanosheets composite, *P* is the volume fraction of Ti_3_C_2_T_x_ MXene nanosheets, *Pc* is the percolation threshold, *β* is the conductivity exponent which is approximately 1.3 and 2.0 for three-dimensional and two-dimensions randomly distributed nanocomposites, respectively, in the percolation model [32]. The density of the Ti_3_C_2_T_x_ MXene nanosheets is ~5 greater than the EP polymer. Therefore, we calculate that the conductivity exponent for EP/Ti_3_C_2_T_x_ MXene nanosheets composite as *β* = 0.84. The Ti_3_C_2_T_x_ MXene nanosheets electrical conductivity is about 24 S/cm which is much higher than conventional non-conductive polymer (~10^−16^ S/cm) and the Ti_3_C_2_T_x_ MXene nanosheets have a large aspect ratio (~100–500), which can be observed in the SEM photograph of the Ti_3_C_2_T_x_ MXene nanosheets. Such an extreme geometry might be a factor contributable to the high electrical conductivity and low conductivity exponent. We can infer that the percolation threshold of EP/Ti_3_C_2_T_x_ MXene nanosheets composite is about 0.28 vol % (0.85 wt %). The percolation threshold of EP/Ti_3_C_2_T_x_ MXene nanosheets composite (0.85 wt %) is higher than that of aligned multi-wall carbon nanotube/EP composite (0.0025 wt %) because aligned CNT by an injection CVD method overcome the obstacle of aggregation and show higher electrical conductivity [32]. However, usually CNT is easy to aggregate in polymer composites, which hamper their high electrical conductivity. So the percolation threshold of our composite is lower than that of usual CNT-based polymer composite [33].

**Figure 8:**
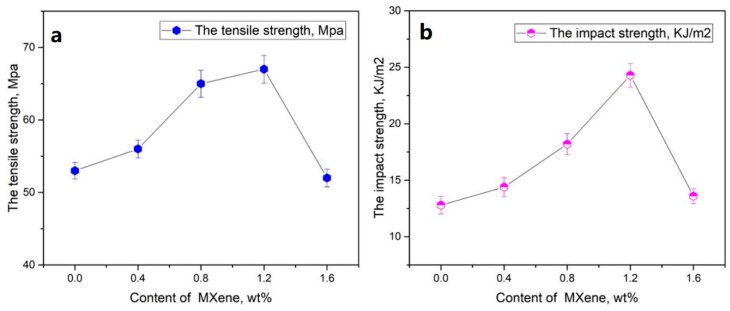
The (**a**) tensile strength and (**b**) impact strength of the EP/Ti_3_C_2_T_x_ MXene nanosheets composite under various contents of the Ti_3_C_2_T_x_ MXene nanosheets.

The mechanical properties (tensile strength and impact strength) of the EP/Ti_3_C_2_T_x_ MXene nanosheets composite are tested with electronic tensile testing machine. The dependencies of tensile strength (a) and impact strength (b) of the EP/Ti_3_C_2_T_x_ MXene nanosheets composite on the content of the Ti_3_C_2_T_x_ MXene nanosheets are shown in Figure 8. Figure 8 shows that the tensile strength and impact strength of the EP/Ti_3_C_2_T_x_ MXene nanosheets composite first increases with the content of Ti_3_C_2_T_x_ MXene nanosheets increasing. When the content of Ti_3_C_2_T_x_ MXene nanosheets increases to 1.2 wt %, the tensile strength and impact strength of composite arrive at the highest value of 66.2 MPa and 24.2 kJ/m^2^, which is better than that in some papers [34]. After that, the tensile strength and impact strength of composite then gradually decreases. It is known that the tensile strength and impact strength of polymer resin is related to the rigidity of the polymer chains. As the content of Ti_3_C_2_T_x_ MXene nanosheets increase, the rigidity of the EP/Ti_3_C_2_T_x_ MXene nanosheets composite increases due to Ti_3_C_2_T_x_ MXene nanosheets that connect many single EP polymer chains by –F, –OH, and –COOH functional groups. However, when the content of Ti_3_C_2_T_x_ MXene nanosheets is more than 1.2 wt %, the EP/Ti_3_C_2_T_x_ MXene nanosheets composite possesses more Ti_3_C_2_T_x_ MXene nanosheets agglomeration, reducing their tensile strength and impact strength.

### 3.4. The Morphology of EP/Ti_3_C_2_T_x_ MXene Nanosheets Composite

**Figure 9:**
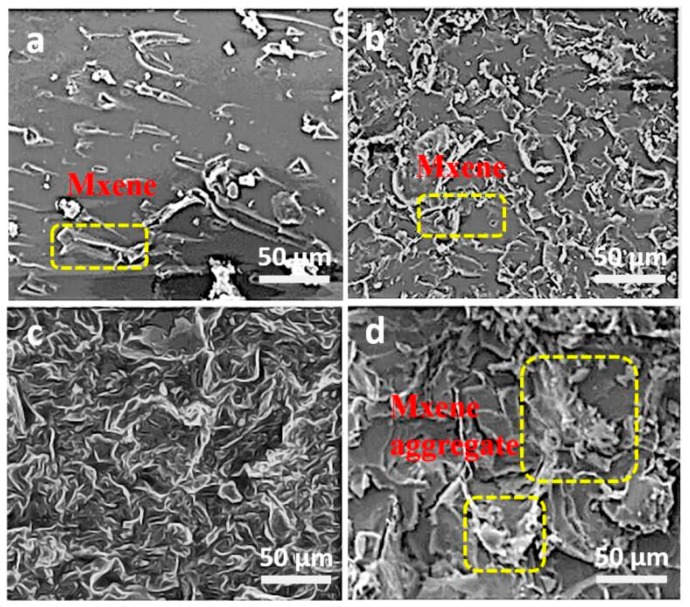
The fracture surfaces of the EP/Ti_3_C_2_T_x_ MXene nanosheets composite containing 0.4 wt % Ti_3_C_2_T_x_ MXene nanosheets (**a**), 0.8 wt % Ti_3_C_2_T_x_ MXene nanosheets (**b**), 1.2 wt % Ti_3_C_2_T_x_ MXene nanosheets (**c**), and 1.6 wt % Ti_3_C_2_T_x_ MXene nanosheets (**d**).

To understand the effect of the content of Ti_3_C_2_T_x_ Mxene nanosheets in NH_2_–CF/MXene/EP composites on mechanical properties, SEM was used to observe the fracture surfaces of EP composites after impact strength test. Figure 9a–d presents the fracture source of EP/Ti_3_C_2_T_x_ MXene nanosheets composite containing 0.4 wt %, 0.8 wt %, 1.2 wt %, and 1.6 wt % Ti_3_C_2_T_x_ MXene nanosheets. The EP/Ti_3_C_2_T_x_ MXene nanosheets composite containing 0.4 wt % Ti_3_C_2_T_x_ MXene nanosheets display comparatively slippery fracture (Figure 9a). The fracture surfaces of EP/Ti_3_C_2_T_x_ MXene nanosheets composite containing 0.8 wt % or 1.2 wt % Ti_3_C_2_T_x_ MXene nanosheets are rougher. In Figure 9d, we can observe that there is apparent agglomeration on the fracture surface of EP/Ti_3_C_2_T_x_ MXene nanosheets composite containing 1.6 wt % Ti_3_C_2_T_x_ MXene nanosheets. The agglomeration of Ti_3_C_2_T_x_ MXene nanosheets could decrease the impact strength of the EP composites. So the SEM results are consistent with the impact strength shown in Figure 8b.

## 4. Conclusions

In summary, a novel ECA based on EP resin filled with Ti_3_C_2_T_x_ MXene nanosheets was prepared by a simple solution blending. Ti_3_C_2_T_x_ MXene is of nanosheet structure, which contains 11.0 wt % C, 69.0 wt % Ti, 11.1 wt % O, 7.8 wt % F, and 1.1 wt % Al. These –F, –OH, and –COOH functional groups make Ti_3_C_2_T_x_ MXene nanosheets homogeneously disperse in EP resin. The electrical conductivity of the EP/Ti_3_C_2_T_x_ MXene nanosheets composite increases gradually as Ti_3_C_2_T_x_ MXene nanosheets content increases, and the percolation threshold of EP/Ti_3_C_2_T_x_ MXene nanosheets composite is 0.85 wt %. The electrical conductivity of EP/Ti_3_C_2_T_x_ MXene nanosheets composite in percolation threshold is 1.81 × 10^−6^ S/m, which is ten orders of magnitude higher than that of the pure epoxy resin (~1.03 × 10^−16^ S/m). The increase in Ti_3_C_2_T_x_ MXene nanosheets content may increase the tensile strength and impact strength of epoxy/Ti_3_C_2_T_x_ MXene nanosheets composite when the content of filler is low. When the content of filler is 1.2 wt %, the properties of epoxy/Ti_3_C_2_T_x_ MXene nanosheets composite are optimum (electrical conductivity of 4.52 × 10^−4^ S/m, tensile strength of 66.2 MPa and impact strength of 24.2 KJ/m^2^). This work presents more applications of MXene-polymer composites.

## Figures and Tables

**Table 1:** Element content of Ti_3_C_2_T_x_ MXene nanosheets.

Element	Atomic %	Weight %
C	27.4	11.0
Ti	40.8	69.0
O	19.0	11.1
F	11.6	7.8
Al	1.2	1.1
